# An imbalance in progenitor cell populations reflects tumour progression in breast cancer primary culture models

**DOI:** 10.1186/1756-9966-30-45

**Published:** 2011-04-26

**Authors:** Simona Donatello, Lance Hudson, David C Cottell, Alfonso Blanco, Igor Aurrekoetxea, Martin J Shelly, Peter A Dervan, Malcolm R Kell, Maurice Stokes, Arnold DK Hill, Ann M Hopkins

**Affiliations:** 1 Department of Surgery, Royal College of Surgeons in Ireland; Dublin, Ireland; 2 Electron Microscopy, UCD Conway Institute, University College Dublin, Ireland; 3 Flow Cytometry, UCD Conway Institute, University College Dublin, Ireland; 4 Division of Gene Therapy and Hepatology, University of Navarra, Bilbao, Spain; 5 UCD Mater Clinical Research Centre, Mater Misericordiae University Hospital, Dublin, Ireland; 6 Pathology, Mater Misericordiae University Hospital, Dublin, Ireland; 7 Surgery, Mater Misericordiae University Hospital, Dublin, Ireland

## Abstract

**Background:**

Many factors influence breast cancer progression, including the ability of progenitor cells to sustain or increase net tumour cell numbers. Our aim was to define whether alterations in putative progenitor populations could predict clinicopathological factors of prognostic importance for cancer progression.

**Methods:**

Primary cultures were established from human breast tumour and adjacent non-tumour tissue. Putative progenitor cell populations were isolated based on co-expression or concomitant absence of the epithelial and myoepithelial markers EPCAM and CALLA respectively.

**Results:**

Significant reductions in cellular senescence were observed in tumour versus non-tumour cultures, accompanied by a stepwise increase in proliferation:senescence ratios. A novel correlation between tumour aggressiveness and an imbalance of putative progenitor subpopulations was also observed. Specifically, an increased double-negative (DN) to double-positive (DP) ratio distinguished aggressive tumours of high grade, estrogen receptor-negativity or HER2-positivity. The DN:DP ratio was also higher in malignant MDA-MB-231 cells relative to non-tumourogenic MCF-10A cells. Ultrastructural analysis of the DN subpopulation in an invasive tumour culture revealed enrichment in lipofuscin bodies, markers of ageing or senescent cells.

**Conclusions:**

Our results suggest that an imbalance in tumour progenitor subpopulations imbalances the functional relationship between proliferation and senescence, creating a microenvironment favouring tumour progression.

## Background

Breast cancer is a heterogeneous disease of considerable social and economic burden. Significant interest surrounds the question whether cancer stem/progenitor cells drive tumour formation [1,2], however it remains to be understood if progenitor analysis has prognostic value in cancer patients. One approach towards interrogating this involves using patient tumour primary cultures to correlate *in vitro *data and clinicopathological information.

Breast progenitor cells are isolated based on expression of markers suggesting capabilities to generate cells of mixed myoepithelial and luminal epithelial lineages [3,4]. Other methods involve isolation of cells positive for aldehyde dehydrogenase (ALDH) activity [5], or ultrastructural identification [6]. Importantly, primary breast cultures retain progenitor/stem cell populations [7].

Using primary cultures from human breast tumour and non-tumour tissue, we sought to define correlations between progenitor cell numbers and clinicopathological or functional indicators of cancer aggressiveness. Our results demonstrate an imbalance between two putative progenitor cell populations in clinicopathologically-aggressive tumours, in conjunction with functional alterations promoting increased proliferation or reduced growth arrest. Taken together, full investigations of progenitor populations in relation to clinicopathological parameters could make an important contribution towards a better understanding of breast cancer progression.

## Methods

### Reagents

Suppliers: trypsin-EDTA, penicillin/streptomycin, penicillin/streptomycin/neomycin, fungizone, Cyquant, X-gal, Alexa-Fluor antibodies (Invitrogen); soybean trypsin inhibitor, collagenase I, hyaluronidase 1-S, DMEM/Ham's F12, bovine insulin, peroxidase-labelled secondary antibodies (Sigma); HMEC, mammary epithelial growth medium (MEGM) kits, foetal bovine serum (FBS, Lonza); glutaraldehyde (Fluka); osmium tetroxide (Electron Microscopy Services). Antibody suppliers: actin, ESA and SMA (Sigma); cytokeratin-19, PE-conjugated CALLA, FITC-conjugated EPCAM, FITC- or PE-conjugated IgG controls (Dako); cytokeratin-18 (Abcam); cytokeratin-14 (Millipore); vimentin and p63 (BD Biosciences).

### Primary cultures

Breast primary cultures were generated from patient lumpectomy/mastectomy samples with informed consent as approved by the Medical Ethics committees of Beaumont Hospital and the Mater Misericordiae Hospital, in accordance with the Declaration of Helsinki. One piece each of tumour tissue and non-tumour margins (Additional file 1) were cultured as described [8]. Tissues were incubated in 10X penicillin/streptomycin/neomycin, minced in DMEM/F12 containing 1X penicillin/streptomycin/neomycin, 10% FBS, 10 μg/ml insulin, 5 μg/ml fungizone, 100U/ml hyaluronidase 1-S, 200U/ml collagenase and rotated for 2 hours/37°C. Supernatants were pelleted, washed and cultured in MEGM. Occasional fibroblast contamination was removed by brief trypsinization (to remove fibroblasts but not underlying epithelial cells), and cultures containing >30% fibroblasts were discarded. In some experiments, primary human mammary epithelial cells (HMEC, Lonza) were cultured in MEGM.

### Breast cell lines

MCF10A and MDA-MB-231 cells (ATCC) grown normally in DMEM-F12, 5% horse serum, 0.5 μg/ml hydrocortisone, 10 μg/ml insulin, 100 ng/ml cholera toxin, 20 ng/ml human recombinant EGF (MCF10A) or DMEM, 10% FBS, 2 mM L-glutamine(MDA-MB-231) were conditioned in MEGM for 2-3 weeks and used in flow cytometry experiments as controls for normal and tumourogenic phenotypes respectively.

### Proliferation assays

Primary cells (5 × 10^3^) were plated in triplicate and harvested after 0, 3 or 6 days. Cyquant solution was incubated on freeze-thawed cells (5 min), and emitted fluorescence detected at 520 nm on a Wallac plate-reader. Fluorescence readings of unknown samples were translated into cell numbers by referring to two separate fluorescence standard curves - one for non-tumour and one for tumour cultures- constructed from known cell numbers (Additional file 2). The slope of each proliferation graph was calculated from the linear regression line using the formula y = mx+c, where m = slope and c = y-intercept.

### Senescence-associated β-galactosidase assays

Primary cells (5 × 10^4^) were plated in duplicate, and stained for senescence-associated β-galactosidase activity [9]. Three brightfield micrographs per condition were captured, and blue senescent cells expressed as a percentage of total cells/field.

### Immunofluorescence staining for epithelial and myoepithelial markers

Primary cells (passage 1-2) grown in chamber slides were fixed in 3.7% paraformaldehyde and immunostained for epithelial (K19, K18, ESA) or myoepithelial (SMA, K14, VIM) markers using DAPI as a nuclear counter-stain. Primary antibodies were omitted in negative controls, and slides visualized on a Zeiss LSM510-meta confocal microscope.

### SDS-PAGE and Western blotting

Confluent primary cultures were harvested in RIPA (20 mM Tris-HCl pH7.5, 150 mM NaCl, 5 mM EDTA, 1% Triton-X100) containing protease and phosphatase inhibitors. Lysates were dounced and 25 μg supernatant subjected to SDS-PAGE and Western blot analysis for K19, K18, VIM and p63.

### FACS analysis of putative progenitor cell populations

Confluent passage 0 primary cells (T25 flask/condition) were trypsinized, blocked in human serum and co-incubated with FITC-conjugated mouse anti-human EPCAM and PE-conjugated mouse anti-human CALLA (4°C/30 min). Negative controls were unlabelled or single-stained with FITC-EPCAM, PE-CALLA, FITC-IgG or PE-IgG. Cells were analyzed on a Beckman Coulter Cyan-ADP and/or an Accuri-C6 flow cytometer. Cells were sorted into CALLA^+^/EPCAM^+^, CALLA^+^/EPCAM^-^, CALLA^-^/EPCAM^- ^or CALLA^-^/EPCAM^+ ^populations on a BD FACSAria cell sorter. Some passage 0 cells were analyzed for activity of the stem cell marker ALDH by Aldefluor assay [5]. Briefly, 2 × 10^5 ^cells were resuspended in assay buffer and incubated with activated substrate or the negative control reagent before analysis.

### Transmission electron microscopy (TEM)

Passage 0 primary cultures or HMECs were fixed with 2.5% glutaraldehyde, processed as described [10] and analyzed on a FEI-Tecnai transmission electron microscope. TEM was also performed on sorted DN subpopulations expanded in 24-well plates.

### Calculations and statistics

Data are expressed as mean ± standard error of the mean. Non-tumour versus tumour results were compared using non-parametric tests and one-tailed unpaired *t*-tests. Population variances were first compared using Instat-3.3.6 to inform the choice of equal/unequal variance between populations. The proliferation:senescence ratio was calculated based upon the data shown in Figure 2B - the linear regression slopes of proliferation graphs and the percentages of senescent cells at the timepoint measured.

## Results

### Primary breast cultures recapitulate the cellular balance of human breast

**Figure 1:**
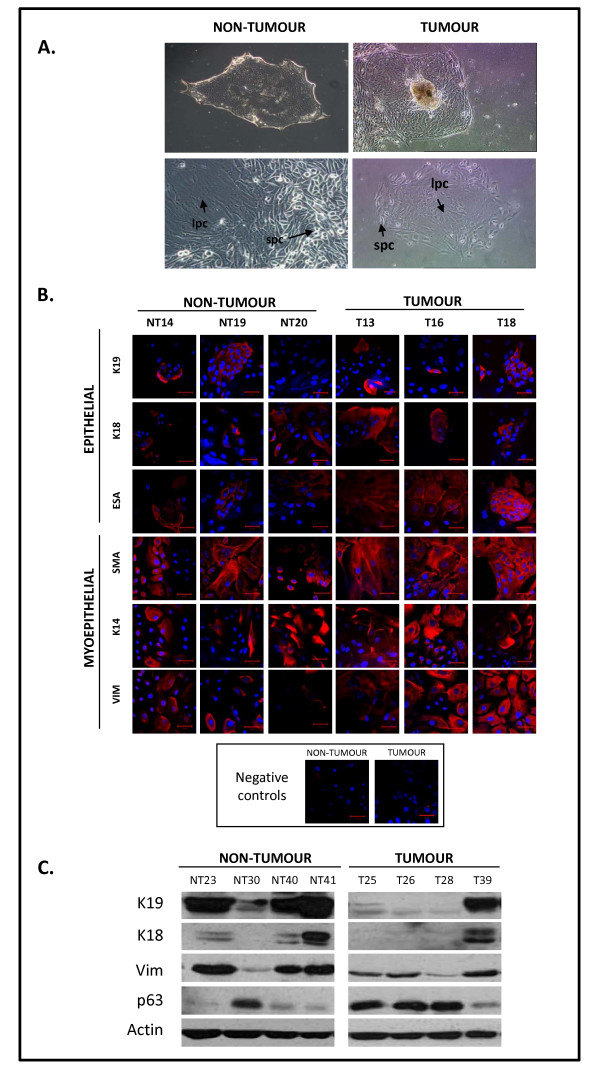
**Characterization of tumour and non-tumour primary cultures**. **A**. Organoid-derived cultures **(A, top panels, 10X magnification) **from both tumour and non-tumour specimens had large polygonal cells **(lower panels, lpc) **surrounded by small polygonal cells **(lower panels, spc, 20X magnification)**. **B**. Representative tumour and non-tumour cultures (passages 1-3) were analyzed for expression of the epithelial markers K19, K18 and ESA and the myoepithelial markers SMA, K14 and vimentin (scale bar 50 μm). **C**. Representative cultures were immunoblotted for expression of epithelial (K19, K18) and myoepithelial (vimentin, p63) markers.

Primary cultures of both non-tumour (NT) and tumour (T) human breast tissue yielded adherent organoids with outwardly-proliferating colonies (Figure 1A, **left**). Two cellular populations were observed - large polygonal cells in colony centres (lpc; Figure 1A, **right**), and small polygonal cells (spc) at the peripheries. Since spc and lpc resembled respectively myoepithelial and luminal epithelial cells, expression of epithelial and myoepithelial markers was examined by immunofluorescence microscopy (Figure 1B). In comparison to the negative control (-ve), cultures were mostly dual-positive for epithelial markers such as K18, K19 or epithelial-specific antigen (ESA) and myoepithelial markers such as K14, vimentin or smooth muscle actin (SMA). Western blot (Figure 1C) detection of K18 was not as sensitive as immufluorescence analysis, since only some of the cultures expressed K18. Interestingly our analysis (Figure 1C) also revealed that 3 out of 4 non-tumour cultures expressed high levels of the epithelial marker K19 and low levels of the myoepithelial marker p63. In contrast, 3 out of 4 tumour cultures expressed low levels of K19 but high levels of p63. Western blotting analysis also confirmed high expression of the myoepithelial marker vimentin.

### Ultrastructural and functional properties of breast primary cultures separate non-tumour and tumour primary cultures

**Figure 2:**
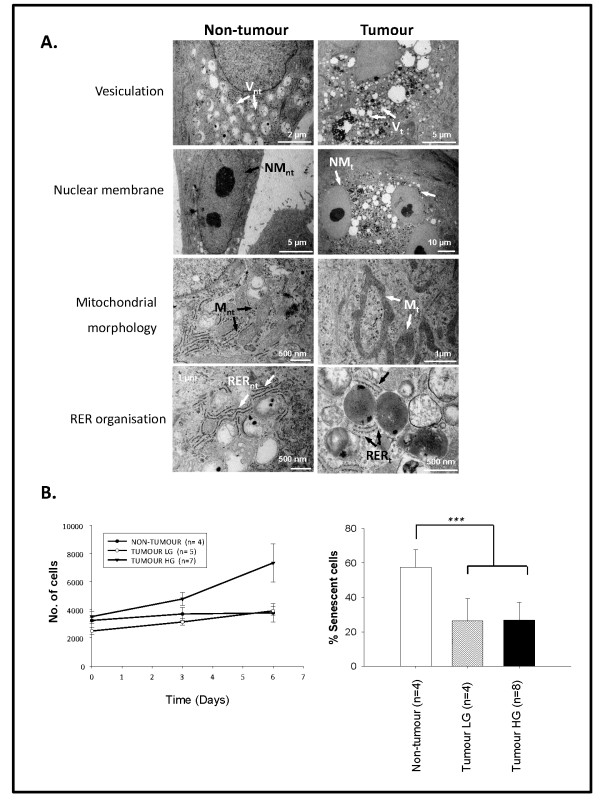
**Ultrastructural and functional differences distinguish non-tumour from tumour primary cultures**. **A**. TEM analysis of non-tumour cells revealed modest numbers of cytoplasmic vesicles (**V**_**nt**_), single nuclei, distinct nuclear double membranes (**NM**_**nt**_), regular mitochondria (**M**_**nt**_) and well-organized RER (**R**_**nt**_). Tumour cells showed abnormal peri-nuclear vesicles (**V**_**t**_), >1 nucleus per cell with thin nuclear membranes (**NM**_**t**_), abnormal mitochondria (**M**_**t**_) and disorganized RER (**R**_**t**_). **B**. Proliferation was enhanced in HG tumour cultures relative to LG tumour cultures or non-tumour cultures **(left)**. Basal senescence, estimated by SA-β-galactosidase staining, was lower in tumour versus non-tumour cultures (**right; **p < 0.001).

Ultrastructural analysis of matched cultures was undertaken to confirm differences between tumour and non-tumour specimens (Figure 2). Firstly, tumour cells were considerably larger than non-tumour cells (~100 μm versus 16 μm respectively along widest axis, data not shown). Extensive abnormal vesiculation patterns were identified in the peri-nuclear regions of tumour versus non-tumour cultures (Figure 2A, V_NT _versus V_T_). Multi-nucleation of tumour cells was frequently observed, in parallel with compromised nuclear membranes (Figure 2A, NM_NT _versus NM_T_). Furthermore, tumour cell mitochondria were abnormal, elongated and occasionally fused (Figure 2A, M_NT _versus M_T_). Finally, non-tumour cells displayed a well-differentiated rough endoplasmic reticulum (RER) while that in tumour cells was fragmented and dispersed (Figure 2A, R_NT _versus R_T_).

We next investigated if morphological differences were accompanied by cell fate differences (Figure 2B). Proliferation abilities were assessed by Cyquant assay on 4 non-tumour cultures and 12 tumour cultures - 5 low grade (LG, grade 1-2) and 7 high grade (HG, grade 3). Values were calculated relative to a standard curve of fluorescence intensity versus known cell numbers (Additional file 2). A significant increase in proliferation was observed in high grade tumour cultures (HG; grade 3) relative to non-tumour or low grade tumour cultures (LG; grades 1-2; Figure 2B, **left**). Since Cyquant proliferation assays quantify all cells rather than just actively-proliferating cells, we performed senescence-associated (SA) β-galactosidase assays [9] to estimate growth arrest (Figure 2B, **right**). Non-tumour cultures had two-fold higher SA-β-galactosidase staining than that in tumour cultures. This was independent of the grade of the originating tumour, and did not reflect an impaired capacity to senesce in response to exogenous stimulation (data not shown).

As the balance between proliferation and senescence is more important than either parameter alone, we examined whether altered proliferation:senescence ratios in breast primary cultures could identify aggressive tumours. The proliferation:senescence relationship was estimated based on proliferation graph slopes and senescence values (Figure 2B). Our data revealed a stepwise increase in proliferation:senescence ratio from non-tumour through LG and finally HG tumours, correlating with a simple model of tumour progression (Table 1).

**Table 1:** Increased proliferation:senescence ratios correlate with tumour progression

	Proliferation:Senescence ratio
Non-tumour (P n = 4; S n = 4)	1.9

Low-grade tumours (P n = 5; S n = 4)	9.5

High-grade tumours (P n = 7, S n = 8)	23.8

where P = proliferation assays, S = senescence assays.

The ratio of proliferation:senescence was calculated for non-tumour, low grade tumour and high grade tumour primary cultures using the slope of proliferation graphs and senescence values from Figure 2B. An increased ratio was observed in the stepwise progression from non-tumour to low grade tumour to high grade tumour categories.

### Alterations in putative progenitor cell subpopulations correlate with aggressive tumours

**Figure 3:**
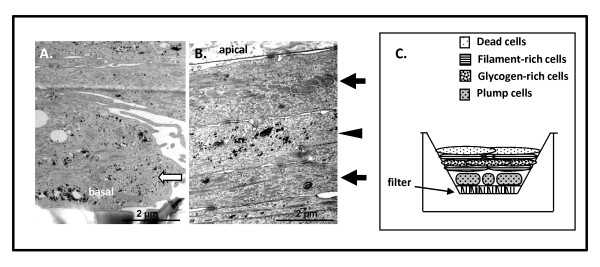
**Ultrastructural identification of putative progenitor cells in primary cultures**. HMEC and tumour primary cultures analyzed by TEM were observed to grow as multi-layers, with basally-located cells having plump morphologies **(a, arrow) **compared to the attenuated morphologies of apically-located cells. Filament-rich cells **(b, arrows) **were layered with glycogen-rich cells **(b, arrowhead)**. A schematic representation of cellular organization is shown in **(c)**.

Since progenitor cells control the generation of new cells in a tissue, we questioned if alterations in progenitor populations could distinguish between aggressive and non-aggressive tumours. Several pieces of evidence suggested the presence of progenitors in primary cultures. Firstly, tumour and non-tumour cultures exhibited epithelial and myoepithelial co-differentiation (Figure 1). Secondly, they expressed the myoepithelial marker p63 (Figure 1C) which is also a progenitor marker [11]. Thirdly, filter-grown cultures had basal electron-lucent, glycogen-rich cells (Figure 3a**arrow**) resembling those described as progenitor/stem cells in mammary duct basal laminae [6]. Apically-located cells were attenuated and squamous-differentiated (Figure 3b**, top arrow**). Layering of dark filament-rich cells (Figure 3b arrows) with light glycogen-rich cells (Figure 3b arrowhead) was observed in all cultures (Figure 3c).

**Figure 4:**
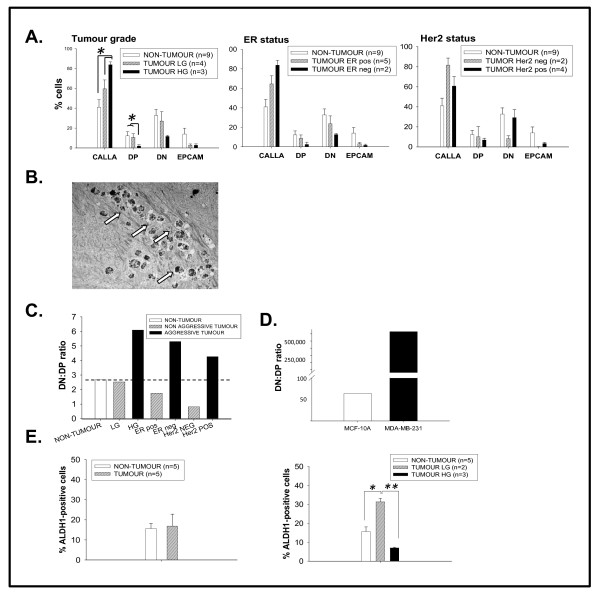
**Isolation of putative progenitor cells from primary cultures and cell lines**. **A**. Breast primary cultures were sorted into CALLA single-positive, EPCAM single-positive, double-positive (DP) or double-negative (DN) populations, and expressed as a percentage of total cells. **B**. TEM analysis revealed a high content of lipofuscin bodies in the DN population sorted from a tumour culture (arrows). **C**. The DN:DP ratio increased in three types of aggressive tumour (high grade, ER-negative or HER2-positive) relative to non-tumour or non-aggressive tumour cultures. **D**. The DN:DP ratio in metastatic MDA-MB-231 cells exceeded that in non-tumourogenic MCF-10A cells. **E**. Activity of the stem cell marker ALDH was similar in non-tumour versus pooled tumour cultures **(left)**, but significantly higher in non-tumour and low grade tumour cultures compared to high grade tumour cultures (p < 0.001; **right)**.

Flow cytometry was used to isolate putative progenitor populations from primary cultures and search for links with clinicopathological evidence of tumour progression. Non-tumour and tumour cultures were analyzed for expression of CALLA (myoepithelial) and EPCAM (epithelial) markers [4,12]. All cultures had highest expression of CALLA and lowest expression of EPCAM single-positive cells, with double-negative (DN) populations exceeding double-positive (DP). Results were grouped according to clinicopathological factors of prognostic relevance, namely tumour grade and expression of ER and HER2 (Figure 4A). The DP population was significantly reduced in aggressive HG relative to LG tumour or non-tumour cultures (p < 0.05), while the CALLA population increased significantly. Both DN and EPCAM populations decreased slightly with increasing grade. Trends were similar in aggressive ER-negative tumour cultures, but not statistically significant. Interestingly, the DN population was increased in aggressive HER2-positive relative to HER2-negative tumours, resembling the larger DN profile of non-tumour cells.

Given DN differences in aggressive HG or ER-negative tumours versus aggressive HER2-positive tumours, we performed ultrastructural analysis on DN populations from one non-tumour and one tumour culture (grade 2 IDC, ER+, HER2+). Although both populations had many similarities (data not shown), unique to the tumour DN population was the presence of abundant lipofuscin bodies (Figure 4B, arrows). These markers of cellular ageing were also observed in unsorted normal and pre-invasive tumour cultures (data not shown).

Since both DN and DP populations are putative progenitor/stem cells [3,4], we questioned whether population ratios better reflected tumour progression than changes in single populations (Figure 4C). Increased DN:DP ratios were observed in all aggressive tumour cultures (HG, ER- or HER2+) relative to non-tumour or non-aggressive tumour cultures. A DN:DP increase was also noted in metastatic MDA-MB-231 cells versus normal MCF-10A cells (Figure 4D). For these experiments, MDA-MB-231 and MCF-10A cells were switched from their normal media and conditioned to grow in MEGM (as used for primary cultures). Although this was not their preferred medium, the cells grew well and we did not observe any morphological differences as a result of media switching (Additional file 3). We also analyzed ALDH activity to estimate progenitor cell numbers. A low percentage of cells were ALDH-positive (Figure 4E, left). However ALDH activity in LG tumour cultures was significantly higher than that in non-tumour cultures (Figure 4E, right). Interestingly, ALDH activity dropped significantly from LG to HG cultures, to lower than that in non-tumour cultures (p < 0.001). This mirrored observed reductions in both DP and DN populations in HG versus LG tumour cultures (Figure 4A).

## Discussion

Intriguing recent work has suggested that immunohistochemical profiling of breast tumours for cancer stem cell populations may have prognostic value [13]. To probe at a cellular level the relationship between progenitor cells and clinicopathological indicators of breast cancer progression, we isolated primary cells from tumour and non-tumour tissue and cultured them in serum-free medium [14]. Although many isolation methods and media formulations have been described over the years, we chose this method because it allowed us a high yield of cells from small tissue samples and because the commercially-available medium offered advantages of consistency and reproducibility relative to self-made medium. Using these culture conditions, most cultures presented two cell-type populations as described [7,15,16], namely large and small polygonal cells which are presumptive epithelial and myoepithelial cells respectively. A relatively crude isolation approach which allows retention of multiple cellular populations may offer advantages over isolation approaches in which cells are purified to homogeneity, since a mixed cell population better recapitulates the cellular balance of tumours *in vivo*.

Myoepithelial marker expression was found to dominate over luminal epithelial expression, consistent with observations in HMEC [17,18]. Expression studies have linked myoepithelial and mesenchymal/basal-like phenotypes; the latter associated with poor patient prognosis [19]. While some studies favour separate media formulations [20], our ultrastructural data suggested that MEGM supported separate growth of non-tumour and tumour populations. For example, malignant characteristics including abnormal vesiculation, branched mitochondria, poorly-developed RER and multi-nucleation were observed only in tumour cultures.

Mesenchymal/basal-like phenotypes also promote progenitor growth and tissue regeneration [21]. The expression of the myoepithelial marker p63 was recently described to be involved in the development of stratified epithelial tissue such as that of the breast, and it has been associated with the presence of progenitor cells and tumour progression [11]. Interestingly, most of our non-tumour cultures expressed the luminal epithelial marker K19, but low levels of the myoepithelial (and progenitor) marker p63, while tumour cultures conversely expressed low levels of K19 and high levels of p63. These data may suggest that non-tumour cultures are enriched in more differentiated cells (K19-positive) than tumour cultures which may be less differentiated and more enriched in multipotent or non-specialized cells (p63-positive) [22]. While K14/K18 are generic markers for discerning epithelial versus myoepithelial cells, K19/p63 are considered to discriminate more differentiated/specialized cells versus non differentiated/specialized cells [11,18,23]. In addition, CALLA/EPCAM have been described to better detect progenitor populations [12]. In fact, we used CALLA and EPCAM as myoepithelial and epithelial markers to subdivide cultures into terminally-differentiated or undifferentiated (putative progenitor) populations. Both populations, double positive (DP) and double-negative (DN) for these markers have been described as putative progenitor cells [3,4]. Our cultures had large DN populations and highest expression of myoepithelial markers, in accordance with other reports [12].

We sought to correlate subpopulation changes with tumour clinicopathological parameters, and observed decreased DP populations in aggressive tumours of high grade or ER negativity. ALDH activity was also reduced in HG tumours, an interesting fact since ALDH expression has been correlated with poor prognosis in breast cancer [5,24] - although the opposite has been reported in ovarian cancer [25]. However we did observe increased ALDH activity in LG tumours relative to non-tumour cultures. Taken together, our results could suggest that DP, DN and ALDH-positive populations are progenitor cells lost from aggressive HG or ER-negative tumours. Perhaps such progenitor cells generate fully-differentiated cells in normal tissue, and their loss could favour undifferentiated phenotypes in aggressive tumours. The DN population was also lower in aggressive HG or ER-negative tumours, but not in aggressive HER2-positive tumours. If individual cells over-expressing HER2 are indeed tumour-initiators [26], our DN results could represent a progenitor population associating with HER2 expression.

DN and DP populations have been described as slightly different putative progenitor/stem cell populations; with DN representing an undifferentiated population while DP represents a multipotent population [4,12]. Since in normal tissue the balance between these 2 populations is tightly regulated, we wondered if the balance is disrupted in malignant phenotypes and may be a marker of tumour progression. Thus in an attempt to mathematically reflect this balance, we calculated the ratios between DN and DP subpopulations. Importantly, we show that a DN/DP imbalance (in the form of increased DN:DP ratios) identifies all three types of aggressive tumour, namely HG, ER-negative or HER2-positive. The abundance of lipofuscin bodies, markers of cellular ageing, in tumour DN populations is an interesting point. Since premature senescence was reduced in tumour versus non-tumour cultures, we speculate that tumour DN populations represent undifferentiated cells capable of senescing, and that DN reductions in aggressive HG or ER-negative tumours suggest loss of an endogenous tumour-suppressive mechanism.

Interestingly, we did not observe DN reductions in HER2-positive cultures. However elevated HER2 can drive premature senescence [27], and high DN:DP ratios better identify aggressive tumours than DN changes alone. Thus loss of a putative pro-senescence (DN) "normal" population is unlikely to drive tumour progression unless proliferation is high. Any pro-senescence (anti-tumourogenic) effects of HER2 could be outweighed by the pro-proliferative effects of HER2 [28]. Our study has illustrated a stepwise increase in proliferation:senescence ratios through non-tumour, LG and HG tumours. The proliferation:senescence balance is an important determinant of tumour progression, dormancy or regression. If the DN:DP ratio estimates this, it could have prognostic value. Although progenitor isolation using markers will never recapitulate the complexity of these plastic and diverse cellular populations, our study nonetheless illustrates that marker studies can yield important insights into clinical samples.

## Conclusions

**Figure 5:**
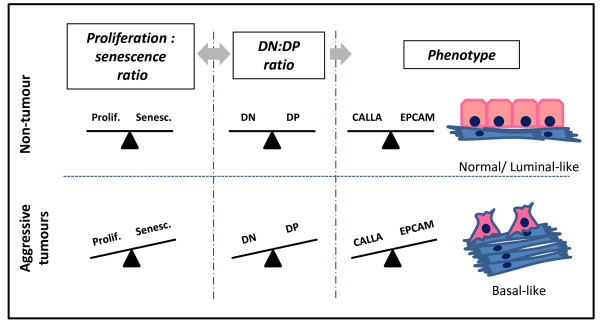
**Progenitor imbalance model**. A normal phenotype likely requires a fine balance between different progenitor populations (DP and DN). In normal cells, a balance between proliferation and senescence interplays with a balance between these putative progenitor populations. This promotes regulated generation of differentiated cells. In aggressive tumours, increased proliferation and decreased senescence influences the equilibrium between different progenitor populations. This may alter the differentiated/undifferentiated cell balance, promoting basal-like phenotypes associated with tumour progression.

We have reported reduced senescence in tumour versus non-tumour breast primary cultures, and stepwise increases in the proliferation:senescence ratio with increasing tumour grade. Isolation of putative progenitor subpopulations revealed a novel correlation between increased DN:DP ratios and clinicopathological indicators of aggressive tumours (HG, ER-negativity or HER2-positivity). Our data suggest that progenitor population imbalance could promote tumour progression by altering the relationship between proliferation and senescence (Figure 5). Future investigations relating clinicopathological factors to alterations in progenitor cell populations may be valuable in dissecting mechanisms associated with progenitor-driven breast tumour progression.

## Abbreviations

MEGM: mammary epithelial growth medium; HMEC: human mammary epithelial cells; DCIS: ductal carcinoma *in situ*; IDC: invasive ductal carcinoma; LC: lobular carcinoma; ITLC: invasive tubular lobular carcinoma; SA-β-gal: senescence-associated β-galactosidase; ER: estrogen receptor; PR: progesterone receptor; ESA: epithelial-specific antigen; SMA: smooth muscle actin; VIM: vimentin; CALLA: common acute lymphoblastic leukaemia antigen; EPCAM: epithelial cell adhesion molecule; DP: CALLA & EPCAM double-positive; DN: CALLA & EPCAM double-negative; HG: high grade; LG: low grade; ALDH: aldehyde dehydrogenase; TEM: transmission electron microscopy; K14: cytokeratin-14; K18: cytokeratin-18; K19: cytokeratin-19.

## Competing interests

The authors declare that they have no competing interests.

## Authors' contributions

SD and AMH conceived and designed the study, analyzed and interpreted the data, drafted the manuscript and revised it. SD performed most of the experimental work, with assistance from LH (primary culture generation), IA (senescence assay set-up), DCC (electron microscopy) and AB (cell sorting). DCC, AB and ADKH contributed to the interpretation of the results. ADKH, PAD, MJS, MS and MRK contributed to patient selection, sample acquisition and clinical interpretation. All authors read and approved the final manuscript.

## Supplementary Material

Additional file 1 **Primary culture patient information**. Click here for file

Additional file 2 **Proliferation assay standard curves for tumour and non-tumour cultures**. Two non-tumour and two tumour cultures were used to generate standard curves to calculate numbers of cells from fluorescence values obtained at different time points of the Cyquant proliferation assays. Click here for file

Additional file 3 **MEGM medium does not alter the morphology of MCF-10A and MDA-MB-231 cells**. MCF-10A and MDA-MB-231 cells were cultured for 15 days in MEGM or their standard serum-positive media, and imaged by phase contrast microscopy. No overt morphological differences were observed in either cell type after the media was switched. Click here for file
