# Supplementary material for: An imbalance in progenitor cell populations reflects tumour progression in breast cancer primary culture models
Source: J Exp Clin Cancer Res. 2011 Apr 26;30(1):45. doi: 10.1186/1756-9966-30-45 (PMC3094256; doi:10.1186/1756-9966-30-45)
Supplement: Additional file 1 — Primary culture patient information. [file 1756-9966-30-45-S1.PDF]

Non-tumour #	Age	Adjacent Tumour Type	Grade	ER	PR	HER2	Assays
NT11	41	IDC	3	pos	pos	neg	S
NT14	52	IDC	3	pos	pos	neg	P,S,IF
NT15	72	IDC	2	pos	pos	neg	P,S
NT19	85	IDC	3	pos	N/A	N/A	IF
NT20	53	IDC	1	neg	neg	neg	S,IF
NT22	47	IDC	3	pos	neg	neg	P
NT23	45	IDC	1	pos	pos	neg	W
NT27	65	IDC	2	pos	neg	neg	P
NT30	47	IDC	1	pos	pos	neg	W,FC
NT32	50	IDC	2	pos	neg	pos	FC
NT33	40	DCIS	1	pos	neg	neg	FC
NT35	33	IDC	2	pos	pos	neg	FC
NT36	37	IDC	3	neg	neg	neg	FC
NT37	43	DCIS	1	pos	pos	neg	FC
NT38	52	IDC	3	pos	pos	neg	FC
NT40	59	IDC	3	N/A	N/A	N/A	W
NT41	39	IDC	3	pos	pos	neg	W
NT43	48	IDC	3	neg	neg	neg	FC
NT46	41	IDC	2	pos	pos	pos	FC

Tumour #	Age	Tumour Type	Grade	ER	PR	HER2	Assays
T1	41	IDC	3	pos	pos	pos	S
T2	60	IDC	2	pos	pos	neg	S
T3	39	IDC	3	pos	pos	pos	P,S
T4	N/A	IDC	1	pos	pos	neg	S
T5	45	IDC	3	pos	pos	neg	P,S
T6	56	IDC	3	neg	neg	pos	S
T7	36	IDC	3	neg	neg	neg	P,S
T8	38	IDC	3	pos	pos	neg	S
T9	58	IDC	1	pos	pos	N/A	S
T10	53	IDC	2	neg	neg	neg	P
T11	41	IDC	3	pos	pos	neg	P,S
T13	50	IDC	3	pos	pos	pos	IF
T14	52	IDC	3	pos	pos	neg	P,S
T15	72	IDC	2	pos	pos	neg	P,S
T16	51	IDC	2	neg	pos	neg	P,IF
T17	60	IDC	3	pos	neg	pos	P
T18	49	IDC	2	pos	pos	neg	IF
T25	52	IDC	1	pos	pos	pos	P,W
T26	44	IDC	3	pos	pos	neg	P,W
T28	49	IDC	2	pos	neg	neg	W,P
T29	65	IDC	1	pos	pos	neg	FC
T31	50	LC	2	pos	pos	neg	FC
T32	50	IDC	2	pos	neg	pos	FC
T33	40	DCIS	1	pos	pos	neg	FC
T34	40	IDC	3	pos	pos	neg	FC
T36	37	IDC	3	neg	neg	neg	FC
T37	43	DCIS	1	pos	pos	neg	FC
T39	47	IDC	2	pos	pos	neg	W
T44	47	IDC	3	neg	neg	neg	FC
T45	47	IDC	3	pos	neg	pos	FC

NT=non-tumor tissue; T=tumor tissue; DCIS=ductal carcinoma *in situ*; IDC=invasive ductal carcinoma; LC=lobular carcinoma; N/A=not available; P=proliferation; S=senescence; IF=immunofluorescence; W=Western blotting; FC=flow cytometry
