# Supplementary figures and images for: An imbalance in progenitor cell populations reflects tumour progression in breast cancer primary culture models
Source: J Exp Clin Cancer Res. 2011 Apr 26;30(1):45. doi: 10.1186/1756-9966-30-45 (PMC3094256; doi:10.1186/1756-9966-30-45)

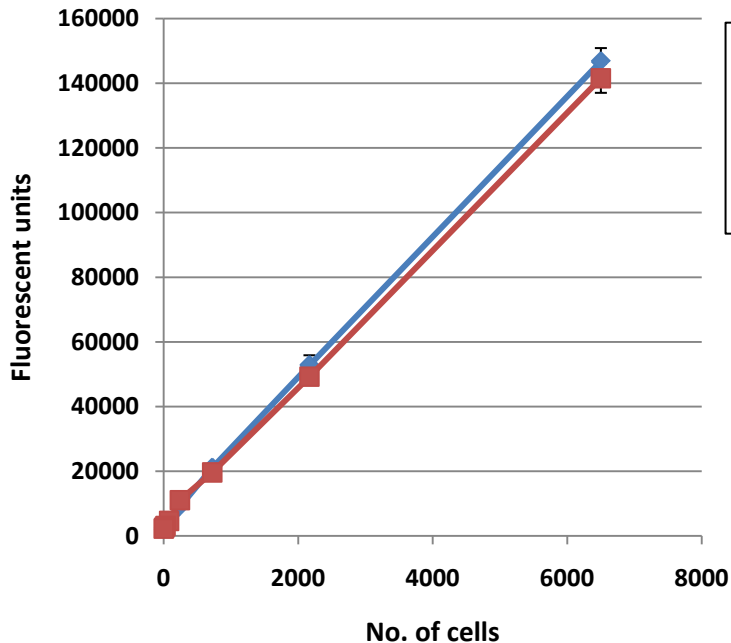

| $y = mx + c$   |               |
|----------------|---------------|
| Non-Tumour     | Tumour        |
| $m = 22.2$     | $m = 21.3$    |
| $c = 3181.7$   | $c = 3326.1$  |
| $r^2 = 0.9995$ | $r^2 = 0.999$ |

—◆— Non-Tumour (n=2)  
—■— Tumour (n=2)

Supplement: Additional file 2 — Proliferation assay standard curves for tumour and non-tumour cultures. Two non-tumour and two tumour cultures were used to generate standard curves to calculate numbers of cells from fluorescence values obtained at different time points of the Cyquant proliferation assays. [file 1756-9966-30-45-S2.PDF]

Serum-rich medium

MEGM medium  
(serum-free)

MCF-10A

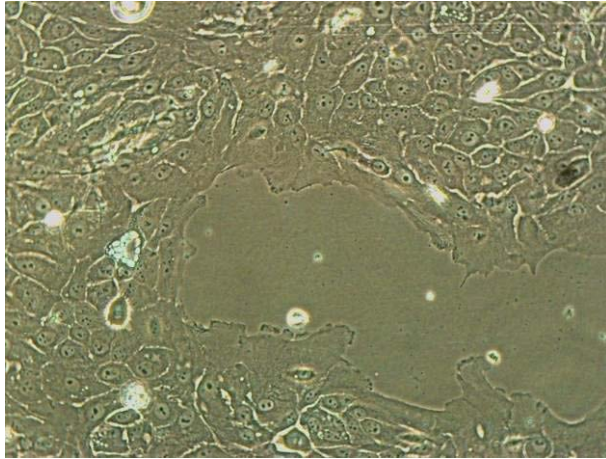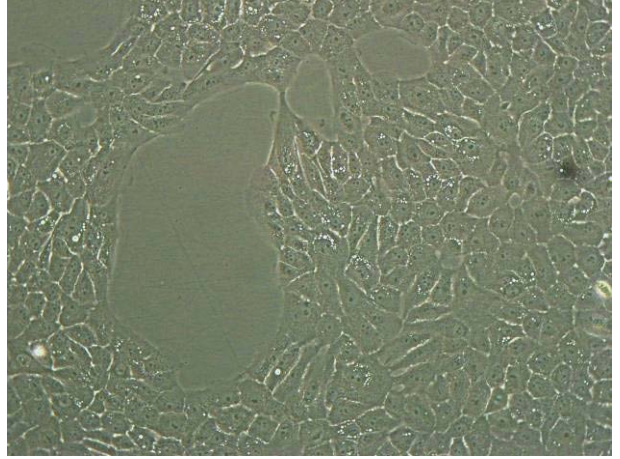

MDA-MB-231

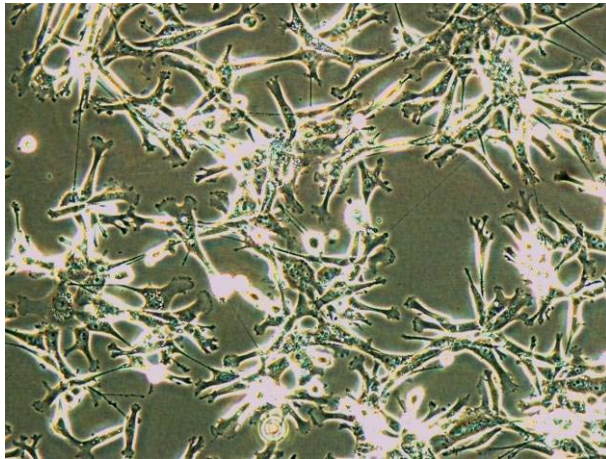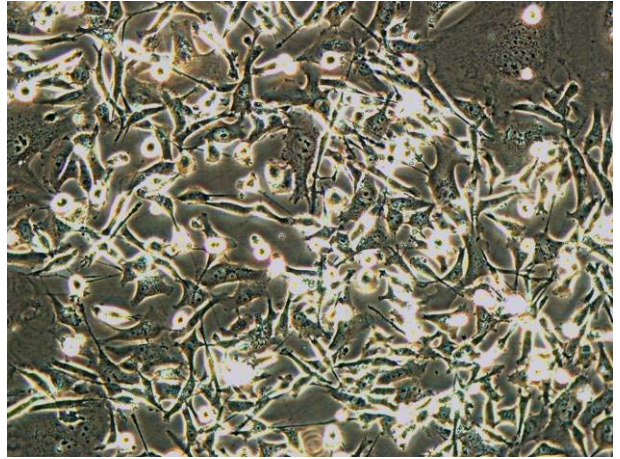

Supplement: Additional file 3 — MEGM medium does not alter the morphology of MCF-10A and MDA-MB-231 cells. MCF-10A and MDA-MB-231 cells were cultured for 15 days in MEGM or their standard serum-positive media, and imaged by phase contrast microscopy. No overt morphological differences were observed in either cell type after the media was switched. [file 1756-9966-30-45-S3.PDF]
